# Microstructure and Mechanical Properties of Hot- Rolled and Cold-Rolled Medium-Mn TRIP Steels

**DOI:** 10.3390/ma11112242

**Published:** 2018-11-11

**Authors:** Chunquan Liu, Qichun Peng, Zhengliang Xue, Shijie Wang, Chengwei Yang

**Affiliations:** 1 The State Key Laboratory of Refractories and Metallurgy, Wuhan University of Science and Technology, Wuhan 430081, China; xuezhengliang@wust.edu.cn (Z.X.); wangshijie1994@163.com (S.W.); 2 Key Laboratory for Ferrous Metallurgy and Resources Utilization of Ministry of Education, Wuhan University of Science and Technology, Wuhan 430081, China; 3 Wuhan Baosteel Central Research Institute, Wuhan 430083, China; ycw258@baosteel.com

**Keywords:** medium Mn steel, mechanical properties, TRIP steel, austenite stability, fracture

## Abstract

This study investigated the microstructure and mechanical properties of hot-rolled and cold-rolled medium-Mn transformation-induced plasticity (TRIP) steel. The experimental steel, processed by quenching and tempering (Q & T) heat treatment, exhibited excellent mechanical properties for hot-rolled and Q & T steels (strength of 1050–1130 MPa and ductility of 16–34%), as well as for cold-rolled and Q & T steels (strength of 878–1373 MPa and ductility of 18–40%). The mechanical properties obtained after isothermal holding at 775 °C for one hour for cold-rolled/Q & T steel were superior to that of hot-rolled/Q & T steel. Excellent mechanical properties were attributed to the large amount of retained austenite, which produced a discontinuous TRIP effect. Additionally, the differences in mechanical properties correlated with the morphology, stability and content of retained austenite. The cold-rolled sample, quenched from 650 °C (CR 650°C) had extensive TRIP effects in the middle and late stages of the deformation, leading to better mechanical properties. The fracture modes of the hot-rolled sample, quenched from 650 °C, and the cold-rolled sample quenched from 650 °C, were ductile fractures, resulting in excellent ductility.

## 1. Introduction

Medium-Mn steels (~3–11 wt.% Mn) are a new category of advanced high strength steels (AHSS) that have attracted research interest worldwide in recent years [1,2,3,4,5]. These steels have fine microstructures and contain a large fraction of metastable retained austenite, thus exhibiting excellent strength and elongation. The microstructure of medium-Mn steel is a dual-phase or tri-phase structure composed of metastable austenite and fine ferrite and/or martensite. In the deformation process, the metastable austenite exhibits a transformation-induced plasticity (TRIP) effect or a twinning-induced plasticity (TWIP) effect, which gives the medium-Mn steel excellent plasticity without sacrificing strength. This allows the material to meet the processing requirement for automobile parts with complex structures [5,6,7,8]. Therefore, the amount of retained austenitein medium-Mn steels determine show extensive is the TRIP effect; that is, the transformation of retained austenite into martensite during deformation increases the strength and ductility of steel simultaneously [1,2,3,4,5,6,7,8]. Our previous workon cold-rolled medium-Mn steel revealed that the steel should contain a high fraction of retained austenite, with appropriate stability [9].

It is worth noting that almost all previous studies on medium-Mn steel were limited to the exploration of microstructure and mechanical properties under the same rolling process. For instance, Song et al. reported that 7.75Mn-2.78Al-0.52C wt.% steels processed by cold-rolling exhibited anultimate tensile strength (UTS) of 1090 MPa and a total elongation (TE) of 56.3% [10]. They attributed the excellent performance to the TRIP/TWIP effect, high dislocation density and high fraction of retained austenite. Zhao et al. revealed that 7.9Mn-0.14Si-0.05Al-0.07C wt.% steels processed by warm-rolling showed an excellent UTS of 1600 MPa and TE of 29% [11]. They found that lath-like ferrite and film-like austenite are the key factors in producing excellent mechanical properties. Zhang et al. found that 9.2Mn-1.6Al-0.15C wt.% steels processed by hot rolling had an excellent UTS of 1499 MPa and a TE of 27.7% [12]. They believe that the stability and morphology of retained austenite play an important role in the mechanical properties and work-hardening rate (WHA).

In summary, a higher content of more stable retained austenite is the key parameter for the development of TRIP steel. At the same time, studying the proportion of multiphase structures, such as ferrite and austenite, and their coordinated deformation is the key to controlling induced phase transition. Although the microstructure evolution and mechanical properties of medium-Mn steel obtained by austenite reverted transformation (ART) or intercritical annealing (IA) treatment have been comprehensively and systematically studied in the same rolling process, the reports on different rolling processes and microstructure adjustment methods are limited. Therefore, this paper aimed to systematically analyze the microstructure and mechanical properties of medium-Mn steel obtained by hot rolling and cold rolling processes. Meanwhile, the austenite stability, strain hardening behavior, strength-plasticity mechanism and mechanical fracture morphology of medium-Mn steel in two rolling processes were compared.

## 2. Experimental Procedure

**Figure 1:**
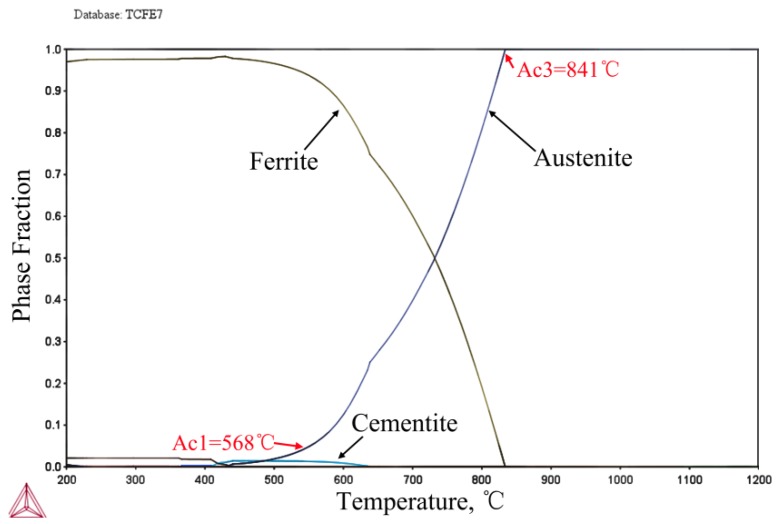
Equilibrium phase fractions of ferrite, austenite and cementite as a function of temperature.

**Figure 2:**
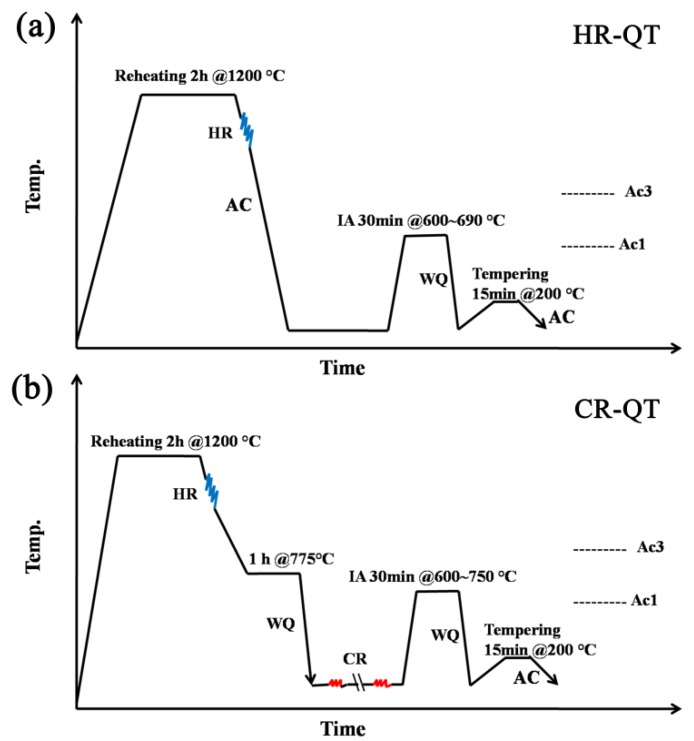
Graph of the thermo-mechanical treatment of quenching and tempering (Q&T) process used (**a**) for the hot-rolled and quenching and tempering(HR-QT) steels, (**b**) for the cold-rolled and quenching and tempering(CR-QT) steels (AC: air cooling, IA: intercritical annealing, WQ: water quenching).

A 15 kg experimental TRIP ingot was prepared via a vacuum induction furnace with a nominal composition of 0.12C-5Mn-1Al-0.2Mo-0.05Nb (wt.%). The ingotwas cast into rods 30 mm × 50 mm in size and then homogenized at 1200 °C for at least 100 min. Afterwards, the ingot was hot-rolled into strips of 3.6mm in thickness. Finally, 44% of reduction was done to obtain cold rolled sheets 2 mm in thickness. The temperatures of A_C_1 and A_C_3 were calculated using the Thermo-Calc^®^ (2017b) and TCFE7 databases, and the values were 568 °C and 841 °C, respectively (see Figure 1). Drawing on previous research results, a process of quenching and tempering heat treatment was adopted in this paper [13]. Figure 2a shows the thermo mechanical process of hot-rolled and quenching and tempering (HR-QT) steels which are mainly subjected to hot-rolling, intercritical annealing and tempering processes. The hot-rolled sheets were first annealed at 600 °C, 625 °C, 650 °C and 690 °C for 30 min respectively, during the quenching and tempering (Q&T) process, followed by quenching and tempering at 200 °C for 15 min. Finally, the samples were cooled to room temperature. The additional heat treatment, prior to cold rolling, reduced the deformation resistance and prevented cracking of the sheets during cold-rolled. The hot-rolled sheets were held at 775 °C for one hour and then quenched to room temperature. Afterwards, the sheets were annealed at 600 °C, 625 °C, 650 °C, 675 °C, 700 °C and 750 °C for 30min, respectively, followed by water quenching to room temperature and tempering at 200 °C for 15 min (as shown in Figure2b).

The plate-shaped tensile specimens were machined to an original width of 12.5 mm and an original gauge length of 50 mm, along the rolling direction. The tensile test was carried out at room temperature and along the axial direction at 3 mm/min on an UTM5305 electronic universal testing machine (Shenzhen, China). After mechanical polishing, the samples were etched in 4 vol.% Nital. Microstructural characterization was observed by a field emission gun scanning electron microscope (Nova 400 Nano SEM) (FEI, Hillsboro, OR, USA) operating at 10 kV and a transmission electron microscope (JEM 2100 F TEM) (JEOL, Tokyo, Japan) operating at 200 kV. The measured Mn content and equilibrium Mn content in different phases (austenite and ferrite) were determined by TEM-EDS (OXFORD, UK) and Thermo-Calc2017b (Thermo-Calc Software, Stockholm, Sweden), respectively. The fracture surfaces of the specimens were observed by a Nova 400 Nano SEM at 18 kV. The content of retained austenite was measured by a Bruker X’Pert Pro powder diffractometer (Bruker, Karlsruhe, Germany). The data acquisition process used a Cu target, with a scan rate of 1°/min and a scan angle of 40°–120°. The content of retained austenite was determined by the integrated intensity of (200)γ, (220)γ, (311)γ, (200)α and (211)α diffraction peaks, and the calculated retained austenite (RA)*V_A_* was calculated using Equation(1) [14,15].

(1) $$V_{A}=\frac{1.4I_{\gamma }}{\left(I_{\alpha }+1.4I_{\gamma }\right)}$$

where *I_γ_* is the integrated intensity of the austenite (200), (220) and (311) crystal plane diffraction peaks and *I_α_* is the integrated intensity of the ferrite (200) and (211) crystal plane diffraction peaks.

## 3. Results

### 3.1. Microstructure Evolution

**Figure 3:**
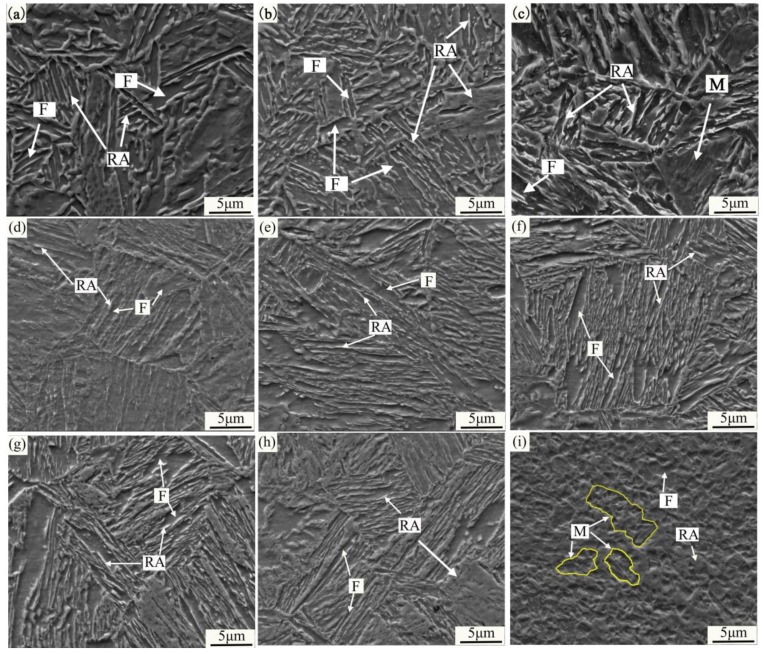
Scanning electron microscope (SEM) micrographs of experimental steel: (**a**) hot-rolled (HR) 625 °C, (**b**) HR 650 °C, (**c**) HR 690 °C, (**d**) cold-rolled (CR) 600 °C, (**e**) CR 625 °C, (**f**) CR 650 °C, (**g**) CR 675 °C, (**h**) CR 700 °C, and (**i**) CR750 °C (RA, retained austenite; F, ferrite; M, martensite).

**Figure 4:**
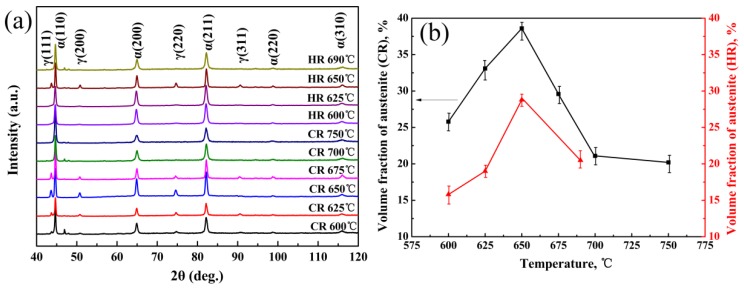
(**a**) X-ray diffraction (XRD) pattern of samples after Q&T treatments and (**b**) the measured austenite fraction with increasing annealing temperature.

Figure 3 shows the SEM micrographs of the hot-rolled and quenching & tempering (HR-QT) and cold-rolled and quenching and tempering (CR-QT) samples. Figure 3a–c describes the microstructure of the HR-QT sample intercritical annealing (IA) at 625 °C, 650 °C and 690 °C, respectively. The microstructure mainly consisted of strip-shaped retained austenite and ferrite when the samples were annealed at 600 °C and 650 °C (abbreviated as HR 600 °C and HR 650 °C). Since the ferrite is elongated and acicular, the austenite is divided into different lengths and thicknesses. From Figure 4b, the lath-shaped austenite broadened with the increasing intercritical annealing temperature, and the volume fraction of austenite increased initially and then decreased. When the sample was annealed at 690 °C (see Figure 3c), the austenite content dropped to a lower level, due to transforming to martensite (see also Figure 4b). Figure 3d–i shows the microstructure of the CR-QT samples IA at 600 °C, 625 °C, 650 °C, 675 °C, 700 °C and 750 °C, respectively. The microstructure mainly consisted of retained austenite and ferrite, when the annealing temperature ranged from 625 °C to 700 °C (abbreviated as CR 625 °C~CR 700 °C). The microstructure of the CR-QT sample consisted of retained austenite, ferrite and martensite when IA was at 750 °C (see Figure 3f). After annealing in the critical region, the quenched martensite was completely reverse transformed into strip-shaped austenite and a small amount of blocked retained austenite (Figure 4g,h), which may be related to the position of austenite nucleation in the process of reverse transformation. Strip austenite readily forms in the lath nucleation of martensite, but bulk austenite is predominant in the original austenite grain boundary or martensite domain. In addition, as the annealing temperature increases, more austenite is obtained by reverse transformation, and the carbon and manganese elements assigned to each austenite grain are relatively reduced; meanwhile, with increasing temperature, austenite grain size increases, which causes the austenite to decrease in stability and be more easily transferred to martensite during cooling.

**Figure 5:**
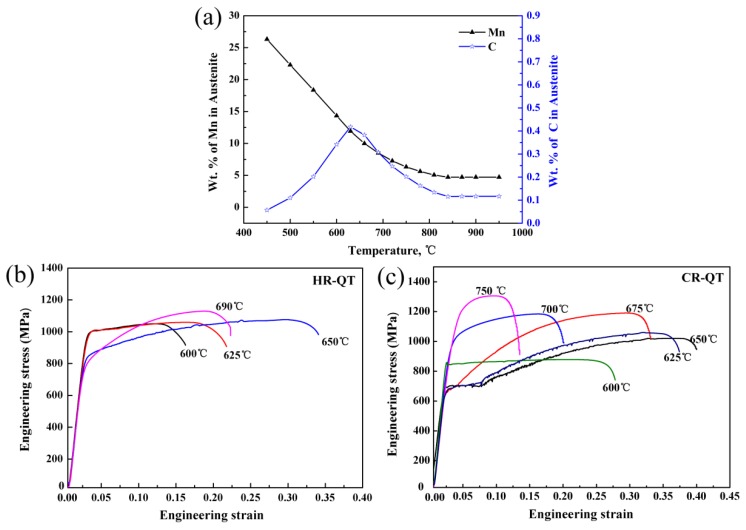
(**a**) Variation of Mn and C equilibrium content of austenite at different annealing temperatures (using Thermo-Calc combined with the TCFE7 database); engineering stress–strain curves of (**b**) HR-QT and (**c**) CR-QT samples.

The measured fractions of RA of the HR-QT and CR-QT samples were obtained from the X-ray diffraction (XRD) patterns, as summarized in Figure 4. Retained austenite in the HR-QT sample had a minimum of ~16 vol.% at the IA temperature of 600 °C. As the IA temperature increased to 650 °C, austenite grain size, as well as the C and Mn contents were reduced, while the austenite content increased to a maximum of about 29 vol.% (see Figure 5a). The reduction in the grain size of austenite resulted in an increase in austenite stability, rendering austenite less likely to transform into martensite. Additionally, the content of austenite significantly increased, due to a low conversion rate to martensite. In the IA temperature range from 600 °C to 750 °C, the fraction of retained austenite in the CR-QT samples increased from ~26 vol.% to a maximum of 39 vol.%, followed by a decrease to a minimum of ~20 vol.%. Interestingly, the hot-rolled sample at the IA temperature of 650 °C had a maximum austenite fraction of about 29 vol.%, while the cold-rolled sample at the IA temperature of 650 °C had a maximum retained austenite fraction of about 39 vol.%. Since the austenite grains in the CR650 °C sample were finer (see Figure 3b,e), the increase in the retained austenite stability hindered its transformation to martensite during the IA process. Kang et al. studied the relationship between the retained austenite fraction of medium Mn steel and the IA temperature [16]. The authors found that the retained austenite fractions should increase first and then decrease with increasing temperature, which is consistent with our results in Figure 4b.

### 3.2. Mechanical Properties

Figure 5a shows the variation in Mn and C equilibrium content of the austenite. The C and Mn contents were greatly affected by the temperature change. As the temperature increased gradually, the C content in the austenite increased first, reaching a maximum value (0.38%) at 660 °C, and then gradually decreased. The content of Mn in austenite decreased with the increasing temperature. Before the temperature reached 800 °C, the Mn content decreased rapidly with increasing temperature, and when the temperature exceeded 800 °C, the Mn content decreased slowly and stabilized. Thus, the main factors affecting the thermal stability of austenite are temperature and changes in C and Mn content. Table 1 summarizes the mechanical properties of the experimental steel, and Figure 5b,c show engineering stress–strain curves of HR-QT and CR-QT samples, respectively. In the HR-QT samples, the ultimate tensile strength (UTS) ranged from 1050 to 1130 MPa and the yield strength (YS) ranged from 956 to 780 MPa. Additionally, the UTS increased with increasing annealing temperature, but the YS decreased with increasing annealing temperature. The HR-QT sample was intercritically annealed to form a soft-phase ferrite with increasing annealing temperature, and the softening effect was related to the low yield strength of the sample. The HR 650 °C sample exhibited excellent mechanical properties, i.e., a TE of 34%, UTS of 1067 MPa and TE× UTS (PSE, product of strength and elongation) of ~36 GPa%. In the CR-QT samples, the UTS ranged from 876 to 1373 MPa, the YS from 659 to 1150 MPa, and the PSE from 21.7 to 41 GPa%. The mechanical strength increased with increasing annealing temperature, while the yield strength decreased first and then increased with the increasing annealing temperature. The softening of the ferrite phase predominantly occurred between 600 °C and 650 °C, resulting in a gradual decrease in yield strength. However, after annealing at temperatures ranging from 675 to 750 °C, a large amount of hard-phase fresh martensite was formed in the process, which contributed to the rebound of the yield strength that has been discussed in our recent research [9]. The CR 650 °C sample exhibited optimum mechanical properties, i.e., a TE of 40%, UTS of 1025 MPa and PSE of 41 GPa%. The reason for the excellent mechanical properties of HR 650 °C and CR 650 °C can be further clarified by studying their work-hardening rate (WHR). There is a caveat, however. Prior to cold-rolling the material was subjected to hot-rolling and isothermal holding at 775 °C for one hour, resulting in a different initial microstructure, compared to the hot-rolled samples. It is unclear whether the resulting properties were due to a difference in initial microstructure or due to the cold-rolling process itself. In order to answer this question, further research is needed. The influence of different initial microstructures and cold-rolling on the mechanical properties of the material requires further study.

## 4. Discussion

### 4.1. Work-Hardening Rate (WHR)

**Figure 6:**
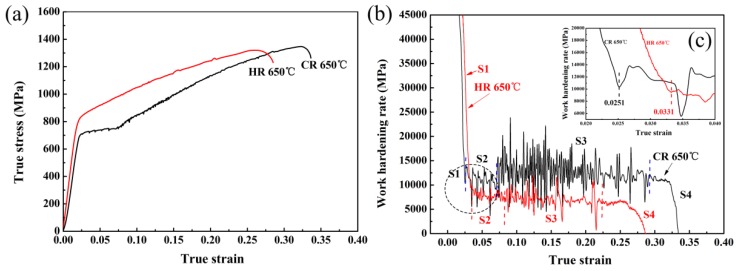
(**a**) True strain-stress curves, (**b**) work-hardening rate (WHR) and (**c**) partial magnification of the area indicated by black dotted circle in (**b**).

Figure 6 shows the true strain–stress curves and WHR of the HR 650 °C and CR 650 °C samples. In the middle-late stages of the strain, the true strain-stress curves of the samples show significant serrations, which should be related to the strain-induced martensite transformation (SIMT) effect (see Figure 6a). Generally, SIMT produces two-fold results: since the formation of martensite counter acts the softening effect caused by stress concentration; on the other hand, due to the hardening effect of the formed hard phase martensite on the material. The competitive effects of these two aspects will change the WHR, which is manifested as a whole or partial rise and fall [17]. In the process of SIMT, concentrated stress is consumed, which is characterized by stress relaxation or a decrease in stress (i.e., the WHR decreased). As the external force continues to increase, the transformed “hard”-phase martensite structure will bear a certain stress, which is characterized by stress concentration. Thus the stress continues to rise (i.e., the WHR increased). The ferrite divides the austenite and changes it from a block to a film of different thickness and length, resulting in austenite with different levels of stability [18]. When SIMT occurs at different stages of retained austenite with different levels of stability, the true stress–strain curve exhibits discontinuous serrations, which can be interpreted as a discontinuous TRIP effect [19,20].

Both the HR 650 °C and CR 650 °C samples exhibit four stages of WHR evolution [21,22]: the WHR decreased rapidly stage (S1); the WHR decreased slowly stage (S2); the WHR fluctuated and increased slowly stage (S3); and the WHR decreased with serrated behavior (S4). In the HR 650 °C sample (see the red line in Figure 6b), the rapid decline of the WHR in the S1 was mainly related to significant deformation of the soft-phase ferrite in the early stage of plastic deformation. Relatively low-stability retained austenite first enhanced the TRIP effect, which offsets the softening effect of the ferrite deformation, resulting in a slow decrease in WHR in S2. With increased strain, some RA with low stability undergoes SIMT to produce a discontinuous TRIP effect that released the stress concentration. This progressive, discontinuous TRIP effect increases the strength of the material, overcoming the softening of the soft phase such as ferrite. This results in a steady rise in the WHR of the S3. In the later stage (S4), the enhanced TRIP effect cannot offset the failure of a large number of plastically deformed structures resulting in a rapid decline in the WHR. In the CR 650 °C sample (see the black line in Figure 6b), the WHR in S1 and S2 did not differ significantly from the WHR in the HR 650 °C sample. The austenite in the CR 650 °C and HR 650 °C sample was transformed at strains of 0.0251 and 0.031, respectively (see Figure 6c). This reveals that the austenite in the HR650 °C sample is relatively difficult to transform, which is consistent with the description of the S1 and S2 WHA changes. The CR 650 °C sample has a longer strain process than the HR 650 °C sample in S3, indicating that the CR650 °C sample produces an extensive discontinuous TRIP effect at this stage and requires a longer time to overcome the softening behavior.

### 4.2. Austenite Stability and Mechanical Fracture Morphology

**Figure 7:**
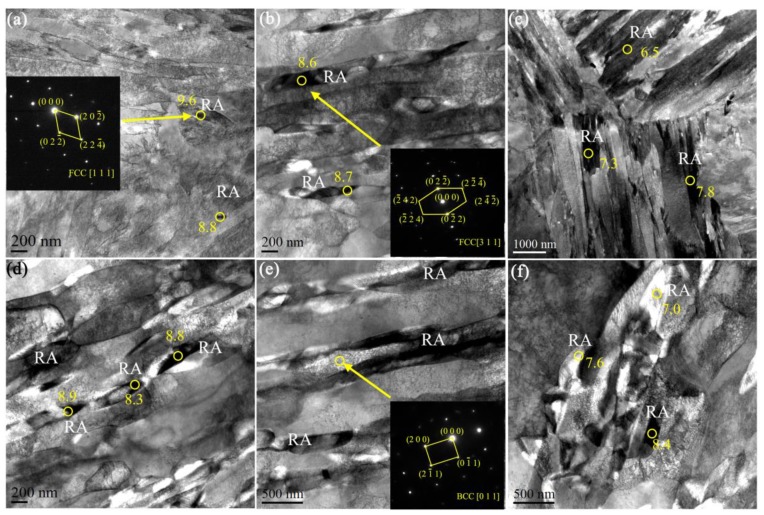
Transmission electron microscope (TEM) micrographs of (**a**)–(**c**) HR 650 °C sample, (**d**)–(**f**) CR 650 °C sample. (**a**) granular austenite grains, (**b**) staking faults and filmy austenite grains, (**c**) blocked austenite grains, (**d**) Mn concentration of the selected austenite grains of the CR 650 °C sample determined by TEM-energy dispersive X-ray spectroscopy (EDS), (**e**) filmy austenite grains, and (**f**) Mn content in austenite grains (RA: retained austenite).

**Figure 8:**
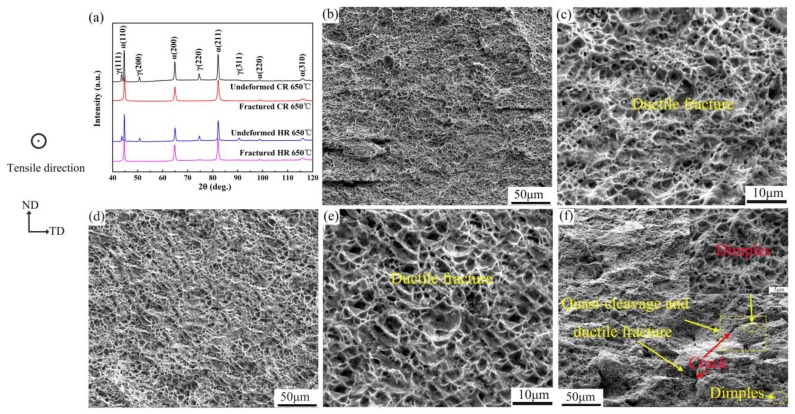
(**a**) XRD patterns of the experimental steel, SEM micrographs of the tensile fracture at different magnifications: (**b**,**c**) HR 650 °C, (**d**,**e**) CR 650 °C, and (**f**) CR 675 °C (ND: normal direction, TD: transverse direction).

García-Mateo et al. showed that too stable austenite does not guarantee better mechanical properties and that retained austenite should have appropriate stability [23]. Previous studies have shown that the stability of austenite depends on various factors such as C/Mn content, grain size and austenite morphology [21]. As shown in Figure 3 and Figure 7, austenite grains can be classified into three types according to its morphology: blocked, filmy, and granular. Transmission electron microscopy (TEM) was used to identify the fine microstructures of the HR 650 °C (Figure 7a–c) and CR 650 °C (Figure 7d–i) samples, as displayed in Figure 7. The granular and lamellar austenite grains can be observed in Figure 7a,b, respectively, and the grain structure was confirmed by indexing the diffraction patterns of the selected area. The granular austenite grains had a size of 80–140 nm, and the film-like austenite grains had a width of 200–350 nm. As shown in Figure 7c, the microstructure morphology consists of blocked austenite. The blocked retained austenite is the least stable, followed by the thin filmy. The finest granularity is the most stable. According to the morphology, we speculate that the HR 650 °C sample has lower austenite stability. However, the speculative result does not match the calculation of the WHR (see Figure 6b). In the CR 650 °C sample, the microstructure mainly consisted of ferrite, granular and filmy austenite grains, which could be seen in Figure 7d–f. The Mn concentration of the selected austenite grains of the CR 650 °C sample was determined by TEM-energy dispersive X-ray spectroscopy (EDS) (Figure 7d,f). Figure 7f shows the filmy austenite and ferrite. Table 2 shows the measured, equilibrium and nominal Mn content in austenite grains of HR650 °C and CR650 °C samples. It can be seen that the average measured Mn content in the HR650 °C sample is higher than that in the CR650 °C sample, and both are lower than the equilibrium manganese content because there is no complete partitioning. Therefore, HR 650 °C sample should have relatively high stability (see also Figure 6b,c). The stability of austenite is further explored using Equation (2) [24]:

(2) $$f_{A}=f_{A0}\exp\left(-k\varepsilon \right)$$

where, *f_A_* is the austenite content at strain ε, *f_A0_* is the original austenite content, and *k* is the calculated austenite stability. The stability of austenite increased with decreasing *k* value [21]. First, the fraction of undeformed and fractured retained austenite in Figure 8a was measured using equation (1), and the *k* value was calculated form Equation (2). The CR 650 °C sample (*k*= 5.4) had a higher *k* value than the HR 650 °C sample (*k*= 4.2), which again proved that the retained austenite in HR 650 °C has relatively high mechanical stability. This reveals that morphology is not a key factor in determining the stability of austenite, and chemical elements in austenite have a greater effect on stability.

Figure 8b–d shows SEM micrographs of the tensile fracture at different magnifications for HR 650 °C, CR 650 °C, and CR 675 °C samples. There are striking differences in the fracture characteristics associated with steel samples of different rolling processes. Figure 8b–e shows that the HR 650 °C and CR650 °C samples have similar dimpled fracture surface. The references have clearly shown that the ductile fracture mode, characterized by the dimple region, effectively alleviates the stress concentration at the crack tip and prevents further crack propagation [25]. The HR 650 °C sample exhibited fine dimples interspersed with deep dimples, while the fracture surface of the CR 650 °C sample contained many deep dimples that were evenly distributed like honeycombs. The CR 650 °C sample had more dimples than the HR 650 °C sample, in terms of size and depth. Both the HR 650 °C and CR 650 °C samples showed ductile fractures (see Figure 8b–e). As shown in Figure 8f, a number of local short cracks are formed at the fracture of the CR 675 °C sample, and the cracks are not overspread, but extend to denser dimple regions and then stop. More evenly distributed small dimple regions were found at the extended tip and both sides of the crack. It can be seen that the dimple region effectively alleviates the stress concentration at the crack tip and prevents further crack propagation. Simultaneously, some quasi-cleavage planes formed at the cracks. Previous studies [26] have shown that the formation of these quasi-cleavages is related to the martensite formed by SIMT. When the crack propagates to the martensite region, the stress concentration is due to the poor deformability of the hard-phase martensite structure, which leads to the rapid expansion of the crack and the formation of the quasi-cleavage plane. In summary, the mechanical fracture mode of HR 650 °C and CR 650 °C samples was considered a ductile fracture, while the mechanical fracture mode of the CR 675 °C sample was of uniformly distributed dimples and a small amount of quasi-cleavage fracture. These fracture patterns reflect the advantages of high ductility. Meanwhile, the size and depth of the dimple of the CR 650 °C sample are higher than those of the HR 650 °C sample, resulting in better ductility of the CR 650 °C than HR 650 °C, which is consistent with our results in Table 1.

## 5. Conclusions

In the present work, the microstructures, mechanical properties, and deformation behavior of experimental steels with different rolling processes were investigated. The microstructure characteristics of HR and CR experimental steels and their effects on mechanical properties and strain-hardening behavior were compared. The main conclusions are as follows: (a) the HR 650 °C sample exhibited excellent mechanical properties, that is, a TE of 34%, UTS of 1067 MPa, and PSE of ~36 GPa%, and the CR 650 °C sample has the optimal mechanical properties, i.e., a TE of 40%, UTS of 1025 MPa and PSE of 41GPa%; (b) after isothermal holding at 775 °C for one hour, the mechanical properties of CR-QT were superior to those of HR-QT; (c) the CR650 °C sample had a more extensive TRIP effect in the middle and late stages of the deformation resulting in better mechanical properties; and (d) the mechanical fracture mode of HR 650 °C and CR 650 °C experimental steel samples is a ductile fracture, resulting in excellent ductility.

## Figures and Tables

**Table 1:** Mechanical properties of the experimental steels.

RAP		YS (MPa)	UTS (MPa)	True UTS (MPa)	YS/UTS	TE (%)	True TE (%)	PSE (GPa%)
HR	600 °C	956	1050	1177	0.84	16	15	16.8
	625 °C	897	1060	1229	0.80	22	20	23.3
	650 °C	812	1067	1360	0.76	34	29	36.2
	690 °C	780	1130	1328	0.69	22	20	24.9
CR	600 °C	832	878	1039	0.95	28	25	24.6
	625 °C	695	1059	1366	0.66	37	32	33.9
	650 °C	690	1025	1350	0.67	40	34	41.0
	675 °C	659	1190	1503	0.55	33	29	39.3
	700 °C	980	1084	1369	0.90	20	18	21.7
	750 °C	1150	1373	1433	0.84	18	13	24.7

RAP, rolling and annealing process; YS: yield strength; UTS, ultimate tensile strength; YS/UTS: yield-to-tensile strength ratio; PSE: UTS×YS.

**Table 2:** The nominal, equilibrium and measured Mn content in austenite (wt.%)

Sample	Nominal content	Equilibrium content	Average measured content
HR 650 °C	5	10.6	8.4 ± 0.2
CR 650 °C	5	10.6	8.0 ± 0.3
